# Editorial: Non-coding RNAs in neurodegenerative diseases

**DOI:** 10.3389/fnins.2023.1241737

**Published:** 2023-07-13

**Authors:** Mingshu Mo

**Affiliations:** Department of Neurology, The First Affiliated Hospital of Guangzhou Medical University, Guangzhou, China

**Keywords:** neurodegenerative diseases, non-coding RNAs, miRNA, circRNA, lncRNA

Neurodegenerative diseases are classed as serial diseases that gradually lose the structure or function of neurons and lead to neurologic dysfunction. They include Parkinson's disease (PD), Alzheimer's disease (AD), Huntington disease (HD), amyotrophic lateral sclerosis (ALS), spinal muscular atrophy (SMA), etc. Different neurodegenerative diseases have different pathological models, clinical manifestations and pathogenesis. Pathological impairments in brains with neurodegenerative diseases mostly develop before patients show clinical symptoms. Discovering new biomarkers to make the early diagnosis is the first step to realize early clinical intervention for these diseases. In the therapy field, the traditional methods for neurodegenerative disease are limited, and the clinical usage of cutting-edge gene therapy still needs a breakthrough. Exploring the potential for other pathogenic mechanisms is a key for advancing knowledge and identifying new directions for potential therapies.

Non-coding RNA (ncRNA) may offer opportunities for early diagnosis and effective clinical intervention for neurodegenerative diseases. NcRNAs are RNA molecules that are not translated to proteins. They contain small-nuclear RNAs (snRNAs), small-nucleolar RNAs (snoRNAs), microRNAs (miRNAs), PIWI-interacting RNAs (piRNAs), small-interfering RNAs (siRNAs), circular RNAs (circRNAs), and long non-coding RNAs (lncRNAs). Most ncRNAs exert their biological functions by regulating the expression of mRNA. Taking miRNA as an example, it could guide the RNA-inducing silencing complex (RISC) to degrade mRNA or hinder its translation by pairing with the target gene mRNA base. MiRNAs have been considered ideal candidates for exploring molecular drugs in clinical research. Meanwhile, miRNA can be protected in exosomes from RNase-dependent degradation, making them stable for detection in plasma or serum samples and can serve as biomarkers for clinical diagnosis. In ALS, the mechanism underlying the functions of multiple miRNAs may have close relationship with the regulation of *TDP-43*, fused in sarcoma gene (*FUS*), and superoxide dismutase 1 (*SOD1*), which may help to explore their clinical applications in the future (Koike and Onodera). A new characteristic expression profile of *miR-485-3p* in PD and aged-matched healthy controls in plasma and plasma-derived small extracellular vesicle (sEV) fractions is presented and valued as a new biomarker in the clinic (Sproviero et al., 2021). Studies on sEVs may elevate the potential diagnostic value of miRNAs in body fluids as biomarkers for disease, especially for neurodegenerative diseases facing serial difficulties in providing brain tissues for detection.

In contrast to traditional RNA with a linear pattern, circRNA molecules have a closed ring structure that cannot be affected by RNA exonucleases, and the expression of circRNA is more stable and difficult to degrade in the body. Some circRNAs act as molecular sponges that bind and block miRNAs to regulate their functions and act as small gene regulators. In neurodegenerative diseases, circRNAs have been found to be involved in the pathogenesis of PD by sponging miRNAs and taking a series of actions, including transcriptive regulation, translation into proteins, protein scaffolding and other pathological mechanisms (Liao et al.). The role of circRNAs also pays attention to cognitive function, as well as their involvement in the occurrence, development, prognosis, and treatment of cognitive-related diseases, including autism, depression, AD (Yu X. et al.) and ischemic stroke (Chen et al.). CircRNAs are also valuable carrier tools or cleaners of miRNAs for gene therapy. For example, *miR-7* can inhibit the gene expression of α*-synuclein* (*SNCA*) in the brain and is involved in α-synuclein pathogenesis in PD (Mo et al., 2017). As miRNA sponges, circRNAs, such as CDR1 antisense (*CDR1as*), can reduce the miR-7 concentration and up-regulated the expression of miR-7–target–genes. By now, how to apply circRNAs in PD treatment is still a challenge (Liao et al.). Related studies may help us to realize the clinical value of circRNAs in neurodegenerative diseases.

LncRNA is an ncRNA longer than 200 nucleotides that plays an important role in mRNA regulation and epigenetics. LncRNA can competitively adsorb special miRNAs through base complementarity as competive endogenous RNA (ceRNA). CeRNAs regulate the loss or decrease of miRNA function and achieve the special control of miRNA–target–gene expression to take biological functions. A new ceRNA network of lncRNA-mediated neuronal apoptosis in hippocampal sclerosis was portrayed to include 10 differentially expressed (DE) lncRNAs, including *CDKN2B-AS1, MEG3*, and *UBA6-AS1*; seven DEmiRNAs, including *hsa-miR-155-5p, hsa-miR-195-5p*, and *hsa-miR-200c-3p*; and three DEmRNAs, including sodium voltage-gated channel alpha subunit 2 (*SCN2A*), dual-specificity tyrosine-phosphorylation regulated kinase 2 (*DYRK2*), and mitogen-activated protein kinase 8 (*MAPK8*) genes (Yu S. et al.). Other special expression profiles of genes and their regulatory mechanisms, including 1,192 circRNAs, 27 miRNAs, and 266 mRNAs, were identified, which may be involved in postoperative neurocognitive disorder (Wu et al.). The undisclosed ceRNA network increases the complexity of regulation of miRNAs. More studies may help to reveal some new mechanisms of neurodegenerative disease related to ceRNA networks in the future.

Recent studies have reported the relationship between neurodegenerative disease and snRNA, snoRNA, and piRNA. SnRNA may combine with protein to form small nuclear heterogeneous ribonucleoprotein particles (snRNPs), which participate in the splicing of pre-mRNAs and produce mature mRNAs. Among them, the U1 snRNP complex is essential in RNA splicing, and its dysfunction may be related to the risk of neurodegeneration, such as AD (Chen et al., 2022). SnoRNA is a type of small non-coding RNA encoded by introns and distributed in the nucleolus of eukaryote cells. SnoRNA has conserved structural elements and mainly takes action in the biological processing of rRNA. Antisense snoRNA can guide rRNA ribomethylation. As an example, *SNORD115* can affect the mRNA editing and splicing of *5-HT2CR* in the brain, and deletion or imprinting of the *SNORD115* gene cluster is a major contributor to features of Prader-Willi syndrome (Huang et al., 2022). PiRNA is an endogenous small interfering RNA that can specifically bind to PiWi-proteins to target invading DNA species, such as transposable elements. Its role in degenerative disease is still unclear.

In the field of novel clinical drugs for neurodegenerative diseases, Nusinersen, as a pioneering antisense oligonucleotide medicine, targets specific mRNA binding to inhibit gene expression, increase the concentration of surviving motor neuron 2 (SMN2) proteins, and successfully intervene the disease process of SMA (Acsadi et al., 2021). NcRNA has a similar targeting mRNA way to realize biological functions and is considered to be another nucleic acid drug as antisense oligonucleotides. Promoting the study of ncRNA in the field of neurodegenerative disease can promote the development of better SMA drugs and discover new targeted drugs for other neurodegenerative diseases.

## Author contributions

The author confirms being the sole contributor of this work and has approved it for publication.
