# Corrigendum: Editorial: Non-coding RNAs in neurodegenerative diseases

**DOI:** 10.3389/fnins.2024.1506380

**Published:** 2024-10-16

**Authors:** Mingshu Mo

**Affiliations:** Department of Neurology, The First Affiliated Hospital of Guangzhou Medical University, Guangzhou, China

**Keywords:** neurodegenerative diseases, non-coding RNAs, miRNA, circRNA, lncRNA

In the published article, there was an error in the Funding statement, where the funding number in “the General Project of Basic and Applied Basic Research of Guangzhou Bureau of Science and Technology (2060206)” was wrong.

The correct Funding statement appears below.

